# Complete Genome Sequence of Bacillus cereus *Sensu Stricto* VKM B-370, Isolated from the Silkworm Bombyx mori

**DOI:** 10.1128/MRA.00386-21

**Published:** 2021-05-20

**Authors:** Emma G. Piligrimova, Rustam M. Buzikov, Olesya A. Kazantseva, Andrey M. Shadrin

**Affiliations:** a Laboratory of Bacteriophage Biology, G. K. Skryabin Institute of Biochemistry and Physiology of Microorganisms, Pushchino Scientific Center for Biological Research of the Russian Academy of Sciences, Federal Research Center, Pushchino, Moscow Oblast, Russia; University of Maryland School of Medicine

## Abstract

Bacillus cereus is a Gram-positive rod-shaped spore-forming bacterium widespread in different environmental niches. Here, we report the complete genome sequence of Bacillus cereus VKM B-370 from the All-Russian Collection of Microorganisms, the host strain for bacteriophages vB_BtS_B83, vB_BcM_Sam46, vB_BcM_Sam112, and Izhevsk.

## ANNOUNCEMENT

Bacillus cereus VKM B-370 was used as the host strain for the propagation of bacteriophages vB_BtS_B83 (1), vB_BcM_Sam46, vB_BcM_Sam112, and Izhevsk (2). The strain was obtained from the All-Russian Collection of Microorganisms (VKM). B. cereus VKM B-370 was plated onto LB agar plates and cultivated at 37°C overnight. A single colony was selected, plated onto a fresh LB agar plate using the streak plate method, and cultivated at 37°C overnight. The procedure was repeated three times, and a single colony from the third plate was grown in 5 ml LB at 37°C overnight with shaking.

Total DNA was isolated using cetyltrimethylammonium bromide (CTAB) precipitation, followed by phenol-chloroform extraction and ethanol precipitation as described by Wilson (short protocol) (3). A MiSeq library was prepared using the KAPA HyperPlus kit (Roche) as recommended by the manufacturer. An Oxford Nanopore library was prepared using the SQK-LSK109 ligation kit and the EXP-NBD104 barcoding kit (Oxford Nanopore Technologies) without DNA shearing or size selection. The library was sequenced on a MinION sequencer using a single FLO-MIN106D R9.4.1 flow cell. Guppy v3.5.0 was used for base calling the Nanopore reads. Illumina and ONT sequencing was performed at BioSpark LLC. A total of 26,164,895 Illumina MiSeq paired-end reads with a length of 101 nucleotides (nt) and 458,060 Oxford Nanopore reads with an average length of 1,084 nt (*N*_50_, 1,890 nt; *N*_90_, 416 nt; maximum read length, 103,689 nt) were generated. Both data sets were used for the genome assembly via the Genome Assembly Service at the PATRIC v3.6.5 website (http://patricbrc.org) (4), using the Unicycler v0.4.8 assembly strategy. Read trimming and quality control were performed using the Trim Galore tool (5) implemented in PATRIC. Assembly polishing was performed using the Racon v1.4.13 (6) and Pilon v1.23 (7) algorithms implemented in PATRIC for long- and short-read data, respectively. All software was used with default parameters, except for the “Trim reads before assembly” parameter, which was set to “true” for the Trim Galore tool. Four circular contigs were obtained (Table 1), suggesting that the genome is represented by a chromosome and three circular plasmids.

**TABLE 1:** Genome characteristics of B. cereus VKM B-370

Contig	Length (bp)	Read coverage^*a*^ (×)	Normalized read coverage (×)	GC content (%)	No. of CDSs	GenBank accession no.
Chromosome	5,387,595	783.0	1.000	35.3	5,216	NZ_CP070339.1
Plasmid pVKMB-370_1	334,064	784.0	1.001	32.9	288	NZ_CP070340.1
Plasmid pVKMB-370_2	86,748	775.9	0.991	33.3	98	NZ_CP070341.1
Plasmid pVKMB-370_3	13,482	4,100.7	5.237	29.7	13	NZ_CP070342.1

a Short-read coverage + long-read coverage.

The chromosome and plasmids were annotated using the NCBI Prokaryotic Genome Annotation Pipeline v4.11 (8). In total, 5,911 genes were identified in the genome, including 5,615 protein-coding sequences (CDSs) excluding pseudogenes, 14 clusters of the 16S, 23S, and 5S rRNA genes, and 106 tRNA genes. According to the PHASTER (9) prediction, two regions of the chromosome, namely, 1 to 25,546 (25.5 kb) and 1,327,661 to 1,376,125 (37.9 kb), may be intact prophages.

The average nucleotide identity analysis based on BLAST (ANIb) was performed on the JSpecies website (10). The ANIb values were higher than 95% for pairwise comparisons between B. cereus VKM B-370 and the following two type strains of the B. cereus group: B. cereus ATCC 14579 (GenBank accession number GCF_006094295.1; 95.29%) and B. thuringiensis ATCC 10792 (GCF_000161615.1; 95.87%). For the other 16 type species (11), the ANIb values were lower than 92%. This result is consistent with the recently proposed changes in taxonomic nomenclature for the B. cereus group (11) and suggests assigning B. cereus VKM B-370 to the B. cereus sensu stricto genomospecies.

### Data availability.

This whole-genome sequencing project has been deposited in GenBank under accession number CP070339 for the chromosome and CP070340, CP070341, and CP070342 for the plasmids. The raw reads are available in the Sequence Read Archive under BioProject accession number PRJNA701373.
